# PsiQuaSP–A library for efficient computation of symmetric open quantum systems

**DOI:** 10.1038/s41598-017-16178-8

**Published:** 2017-11-24

**Authors:** Michael Gegg, Marten Richter

**Affiliations:** Institut für Theoretische Physik, Nichtlineare Optik und Quantenelektronik, Technische Universität Berlin, Hardenbergstr, 36 EW 7-1, 10623 Berlin, Germany

## Abstract

In a recent publication we showed that permutation symmetry reduces the numerical complexity of Lindblad quantum master equations for identical multi-level systems from exponential to polynomial scaling. This is important for open system dynamics including realistic system bath interactions and dephasing in, for instance, the Dicke model, multi-Λ system setups etc. Here we present an object-oriented C++ library that allows to setup and solve arbitrary quantum optical Lindblad master equations, especially those that are permutationally symmetric in the multi-level systems. PsiQuaSP (Permutation symmetry for identical Quantum Systems Package) uses the PETSc package for sparse linear algebra methods and differential equations as basis. The aim of PsiQuaSP is to provide flexible, storage efficient and scalable code while being as user friendly as possible. It is easily applied to many quantum optical or quantum information systems with more than one multi-level system. We first review the basics of the permutation symmetry for multi-level systems in quantum master equations. The application of PsiQuaSP to quantum dynamical problems is illustrated with several typical, simple examples of open quantum optical systems.

## Introduction

In quantum optics and more recently also quantum information research is often centered around how multiple quantum emitters or multi-level systems interact with each other and/or the (photonic) environment. Generally using an open system description is desirable, since dissipation and dephasing are omnipresent. In these systems many body effects produce a rich variety of physical effects but the full quantum description of many emitters usually results in an exponential complexity for numerical treatments. Since exact analytic solutions in such systems are rare and a straightforward numerical treatment of systems of exponential complexity is limited to very small systems alternative methods are necessary.

In a recent publication^1^ we have shown that identical emitters in quantum optical Lindblad master equations result in a permutation symmetry that can be used to reduce the complexity from exponential to polynomial in the number of multi-level systems *N*. In this article we introduce a ready to use computer library for quantum optical master equations for systems of many identical emitters^2^. The permutation symmetry allows to reduce the exponential complexity to polynomial without any approximation. The library is called *PsiQuaSP–Permutation symmetry for identical Quantum Systems Package*. PsiQuaSP allows to exploit permutation symmetry for multi-level systems also including two-levels. For permutation symmetric two-level systems also other code is available^3–5^. For general open quantum systems calculation beside permutation symmetry established, user friendly frameworks^6,7^ exist (also including system bath interactions) and codes for working with parallel solvers^8^. From a theoretical viewpoint, the permutation symmetric method fills the gap for few and intermediate multi-level system numbers *N* left by quantum optical phase space methods such as the positive P representation^9–13^ that cover large *N*–the classical limit. We generalize the density matrix element formulation of ref.^1^ towards a formulation using symmetrized Liouville space states and elementary permutation symmetric Liouville space operators. This treatment is mathematically more general and thus allows for maximal flexibility for the construction of Liouvillians and e.g. observables.

The permutation symmetric approach is exact/non-approximate and non-perturbative, which implies that the method is valid for any permutation symmetric master equation and all parameter ranges. For two-level systems the method has been successfully used by various authors^3,5,10,11,13–23^. Examples for compatible open system setups that can be described are Dicke super- and subradiance^5,22,24–26^, lasers and related devices^19,26^, theoretical toy models such as the open Lipkin-Meshkov-Glick model^27–29^, many particle contributions to STIRAP or coherent population trapping in three-level systems^30^, multi- biexciton cascades in quantum dots^31^ and others. The method allows to study all these systems, including realistic dephasing and dissipation while giving full access to the complete density matrix and thus all information about the system. The mentioned quantum systems are studied in many different contexts including different types of phase transitions^22,29,32–35^, generation of quantum light^31,36^, lasing^19,26,37^, entanglement^18,38–40^, squeezing of collective spins^41^, super- and subradiance^5,22,26,42–44^ and quantum information storage^22,43,45,46^. Furthermore the permutation symmetry was used in the context of quantum tomography of many particle setups^47,48^. Our library allows to directly solve related master equations for moderate multi-level system numbers. This includes the study of quantum many body effects in the presence of dephasing, which was not feasible previously for these systems.

PsiQuaSP enables the setup of the master equation in computer code. The actual numerical solution is entirely handled by PETSc^49–51^ and related packages such as SLEPc^52–54^. These are state-of-the-art packages for efficient sparse linear algebra methods and differential equations. PETSc and SLEPc use MPI distributed memory parallelism. Additionally PETSc provides interfaces to many advanced, external libraries for e.g. specialized linear algebra tools and optimization of parallel performance like MUMPS^55^, SuperLU^56^, METIS/ParMETIS^57^, PTScotch^58,59^ and others. This ensures that PsiQuaSP users can use current and most appropriate algorithms and can directly access the advanced computational sparse matrix methods available through PETSc.

The paper is organized as follows: In Section *Lindblad master equations and permutation symmetry* we give a quick introduction to the permutation symmetry methodology of ref.^1^. Especially we introduce sketches which facilitate the setup of the simulation. In Section *Using PsyQuaSP - Basic structure of the library* we explain the basic design of the library, how it should be used and illustrate the application of the library using a simple two-level system example. More information and further examples can be found in the Supplementary Information. In Section *Template functions versus custom Liouvillians* we give an overview over all ready-made Liouville operator templates in PsiQuaSP and explain how to construct custom types in Section *Building arbitrary Liouvillians*. Finally in Section *Performance* we give a short discussion about the performance of the library.

## Lindblad master equations and permutation symmetry

As stated in the previous section we target Lindblad master equations of collections of identical, indistinguishable multi-level systems. The prerequisite of identical, indistinguishable systems results in the permutation symmetry.

*Notation:* We label the states of the individual multi-level system with integers starting from zero: $\mathrm{|0}{\rangle }_{i}$, $\mathrm{|1}{\rangle }_{i}$, $\mathrm{|2}{\rangle }_{i}$, $\ldots$. $\mathrm{|0}{\rangle }_{i}$ is usually the ground state and the index *i* refers to the individual system. The individual multi-level system is often just referred to as spin. We use general spin matrices describing the individual system/spin according to their Ket and Bra notation:

1 $${\sigma }_{kl}^{i}=|k{\rangle }_{i}\langle l{|}_{i}.$$

The direct product ⊗ of *n* spin matrices *σ*_*kl*_, each referring to another multi-level system is denoted as

2 $${\sigma }_{kl}^{\otimes n}=\mathop{\underbrace{{\sigma }_{kl}^{i}\otimes {\sigma }_{kl}^{j}\otimes \cdot \cdot \cdot }}\limits_{n\,factors\,}\cdot $$

The Liouville space basis for an individual two-level system is formed by four spin matrices, for three-level systems by nine matrices and for general (*d* + 1)-level systems by (*d* + 1)^2^ spin matrices. General collective spin operators are defined as

3 $${J}_{kl}=\sum _{i\mathrm{=1}}^{N}{\sigma }_{kl}^{i},$$

Bosonic operators (e.g. for photons, phonons etc.) are labeled as *b* and *b* ^†^. In the context of two-level systems it is customary to define the ${\sigma }_{z}^{i}=\frac{1}{2}({\sigma }_{11}^{i}-{\sigma }_{00}^{i})$ and ${J}_{z}={\sum }_{i}{\sigma }_{z}^{i}$ operators and also to use the labels +, − instead of 10,01. We do not use this two-level system notation in this report since it is confusing for multi-level systems and our aim is a clear and consistent notation for all types of multi-level systems.

### Examples for master equations

**Figure 1:**
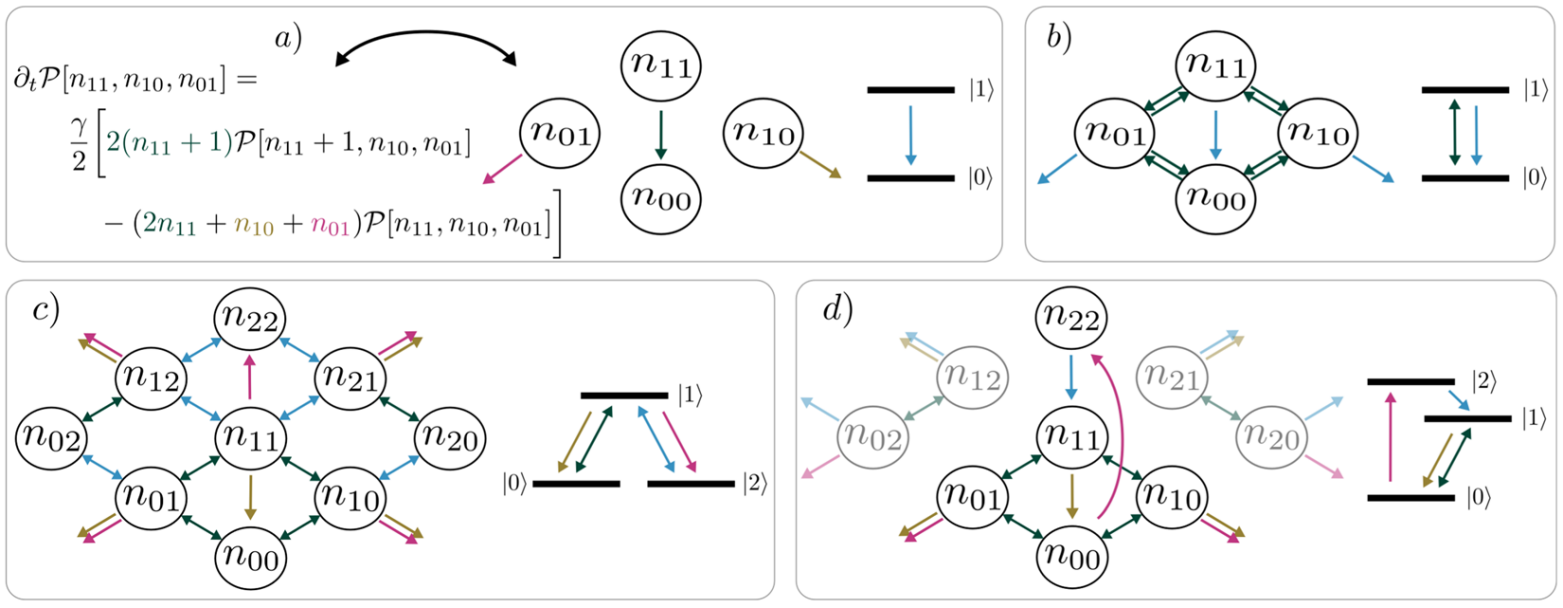
Illustration of the processes of the master equations for two- and three-level systems (right side in **a**–**d**) shows level schemes and left side shows corresponding sketches): (**a**) Translating an equation into a sketch–arrows and corresponding terms have the same color. The green arrow depicts the loss of excitation, states with *n*_11_ + 1 decay into states with increased *n*_00_ until reaching the ground state (i.e. *n*_11_ = 0, *n*_00_ = *N*). The yellow and purple arrows depict the dephasing. The offdiagonal elements (${n}_{10},{n}_{01}\ne 0$) are just dephased. The arrows pointing to the outside indicate loss. (**b**) Open Dicke/Tavis-Cummings model: Emitter-mode coupling part (green arrows) of equation (6) and individual spontaneous emission part, equation (7) (blue arrows). (**c**) Λ-system setup of equations (9), (10): Two different interactions from equation (9) (green,blue) and two different spontaneous emission processes from equation (10) (yellow,purple). (**d**) Three-level laser setup (ref.^1^): Population mechanism through incoherent driving (pink,blue), coupling to the lasing mode (green) and spontaneous emission into nonlasing modes (yellow). Four coherence degrees of freedom (*n*_20_, *n*_21_, *n*_02_ and *n*_12_) are decoupled from the densities.

A general Lindblad equation is defined as^60^

4 $${\partial }_{t}\rho = {\mathcal L} \rho ,$$

where *ρ* is the density matrix and ${\mathcal L}$ is a general, hermitianity and trace preserving Liouville space operator. This operator is sometimes called Liouville super-operator or just Liouvillian. One example for a master equation with permutational symmetry is the open Dicke model, i.e. a set of identical two-level systems coupled to a bosonic mode

5 $${\partial }_{t}\rho =\frac{i}{\hslash }[\rho ,H]+{{\mathscr{D}}}_{1}(\rho )+{{\mathscr{D}}}_{2}(\rho ),$$

with the usual Dicke Hamiltonian^25^ (see Fig. 1b) right)

6 $$H=\hslash {\omega }_{0}{b}^{\dagger }b+\hslash {\omega }_{11}{J}_{11}+\hslash g({J}_{01}+{J}_{10})({b}^{\dagger }+b),$$

and including e.g. the two-level system spontaneous emission and cavity lifetime Lindblad dissipator

7 $$\begin{array}{rcl}{{\mathscr{D}}}_{1}(\rho ) & = & \frac{\gamma }{2}\sum _{i}\mathrm{(2}{\sigma }_{01}^{i}\rho {\sigma }_{10}^{i}-{\sigma }_{11}^{i}\rho -\rho {\sigma }_{11}^{i}),\\ {{\mathscr{D}}}_{2}(\rho ) & = & \frac{\kappa }{2}\mathrm{(2}b\rho {b}^{\dagger }-{b}^{\dagger }b\rho -\rho {b}^{\dagger }b\mathrm{).}\end{array}$$

The setup is permutationally symmetric since the two-level system parameters in this equation, i.e. *ω*_1_, *g* and *γ* are identical for all two-level systems. Exchanging the indices of any two two-level systems results in the same equation. If ultra-strong coupling effects are not present it is possible to treat the interaction Hamiltonian of equation (6) in the rotating wave approximation, resulting in the Tavis-Cummings Hamiltonian^61^

8 $$H=\hslash {\omega }_{0}{b}^{\dagger }b+\hslash {\omega }_{11}\,{J}_{11}+\hslash g({J}_{01}{b}^{\dagger }+{J}_{10}b).$$

All quantum master equations for sets of multi-level systems, where the parameters in the master equation do not depend on the index of the individual multi-level systems show this permutation symmetry. Another example would be a collection of Λ systems, where for instance one transition is interacting with a bosonic mode and the other is driven by an external laser, see Fig. 1c right. In an appropriate rotating frame the Hamiltonian reads

9 $$H=\hslash {{\rm{\Delta }}}_{0}{b}^{\dagger }b+\hslash {{\rm{\Delta }}}_{1}{J}_{22}+\hslash g({J}_{01}{b}^{\dagger }+{J}_{10}b)+\hslash E({J}_{21}+{J}_{12}),$$

where Δ_0_ is the detuning between the 0–1 transition and the cavity mode and Δ_1_ is the detuning between the 1–2 transition and the driving laser. Open system contributions are e.g. spontaneous emission and a finite photon lifetime

10 $$\begin{array}{c}{{\mathscr{D}}}_{1}(\rho )=\frac{\gamma }{2}\sum _{i}\mathrm{(2}{\sigma }_{01}^{i}\rho {\sigma }_{10}^{i}-{\sigma }_{11}^{i}\rho -\rho {\sigma }_{11}^{i}),\quad \quad {{\mathscr{D}}}_{2}(\rho )=\frac{\gamma ^{\prime} }{2}\sum _{i}\mathrm{(2}{\sigma }_{21}^{i}\rho {\sigma }_{12}^{i}-{\sigma }_{11}^{i}\rho -\rho {\sigma }_{11}^{i}),\\ {{\mathscr{D}}}_{3}(\rho )=\frac{\kappa }{2}\mathrm{(2}b\rho {b}^{\dagger }-{b}^{\dagger }b\rho -\rho {b}^{\dagger }b\mathrm{).}\end{array}$$

An analytic solution is only possible for very few of such equations and there are many different approximate schemes to attack this problem: Phase space methods like positive P representation^12,13,62^, limits like single excitation limit^63^ or reductions to the superradiant or general completely symmetric multiplet subspaces^29,64^ and related techniques like Holstein-Primakoff transformation and -approximation^25,64,65^, truncation of the hierarchy of operator expectation values–also called cluster expansion or mean field description^37,66,67^ or, more recently, matrix product state or matrix product operator based truncation schemes explicitly for spin-boson models^68^. There are also non-approximate approaches like quantum trajectory/quantum-jump Monte Carlo^69,70^. All these approaches have their advantages and drawbacks, together they cover a large portion of parameter space described by Lindblad equations in quantum optics. However in the few multi-level system limit, with strong correlations and systems outside the few excitation limit these methods are not well suited. For these applications we believe that the use of the permutation symmetry and its implementation in PsiQuaSP may be advantageous compared to existing methods. Furthermore the exact approach presented in this report can be used to explicitly test the range of validity of other approximate methods.

### Some theoretical details

Exploiting the permutational symmetry of Lindblad equations results in a polynomial complexity in the number of multi-level systems instead of an exponential complexity. This is equivalent to projecting the master equation onto a subspace of special symmetrized Liouville space states. This approach is only valid if the master equation obeys the permutation symmetry. These symmetrized basis states have been introduced and discussed for two-level systems by various authors^1,3,10,11,13–17,19,22^, notably Hartmann called them generalized Dicke states^16^. For multi-level systems the scheme can be derived by explicitly looking at the time evolution of density matrix elements (see refs^1,19^). The possible use of Lie algebraic techniques like Holstein-Primakoff transformation in Liouville space in combination with the permutation symmetry in multi-level system master equations is discussed in ref.^71^.

For a collection of (*d* + 1)-level systems the special symmetrized Liouville space states are given by

11 $$\hat{{\mathscr{P}}}[\{{n}_{kl}\}]=S\underset{k,l\mathrm{=0}}{\overset{d}{\otimes }}{\sigma }_{kl}^{\otimes {n}_{kl}},$$

where $\{{n}_{kl}\}=\{{n}_{dd},{n}_{d(d-\mathrm{1)}},\ldots \}$ is the set of all numbers *n*_*kl*_. The product in equation (11) consists of *N* individual spin matrices, one for each multi-level system. Thus in this direct product each spin is exactly represented by one of the (*d* + 1)^2^ individual spin matrices and *n*_*kl*_ spins are in the same *σ*_*kl*_ state. The ordering in this product is not uniquely determined, there are many permutations that can be written as such a product of spin matrices, characterized by the numbers {*n*_*kl*_}. The symmetrization operator *S*

12 $$S=\sum _{P}\hat{P}$$

creates a sum over all these possible permutations *P* of the spin matrices ${\sigma }_{kl}^{i}$ for a given configuration {*n*_*kl*_}. Here $\hat{P}$ is the permutation operator. This results in an unambiguous definition of totally symmetrized states. Please note that our definition of the symmetrization operator does not contain a normalization factor in contrast to the symmetrization operator usually used for constructing *N* particle boson states. Omitting the normalization makes the method numerically more stable, see ref.^1^.

The number of possible permutations is given by a multinomial coefficient

13 $$(\begin{array}{c}N\\ \{{n}_{kl}\}\end{array})=\frac{N!}{{n}_{dd}!{n}_{d(d-1)}!\ldots {n}_{00}!}.$$

This can be justified as follows: A set of *N* multi-level systems is divided into (*d* + 1)^2^ subsets, one for each individual spin matrix. Then the *n*_*kl*_ are the numbers of elements in these sets and the number of possible realizations is given by the multinomial coefficient equation (13). Please note that this is precisely why this method has a polynomial instead of exponential complexity: Each density matrix element corresponding to one of the permutations in equation (11) is identical to the density matrix elements of all the other permutations. This holds if the master equation has permutation symmetry and the system is prepared in an initial state that obeys permutation symmetry. This requirement is fulfilled if the system is prepared in e.g. the ground or a thermal equilibrium state. Summing over all states that correspond to these identical density matrix elements results in equation (11).

The product in equation (11) contains exactly one spin matrix per multi-level system, this implies

14 $$\mathop{\underbrace{\sum _{kl}{n}_{kl}}}\limits_{m\,summands}=N,\,with\,0\le {n}_{kl}\mathrm{.}$$

This expression determines the number of different basis states and thus the complexity or dimensionality of the problem: The number of possible sets {*n*_*kl*_} that satisfy equation (14) is given by^1^

15 $$(\begin{array}{c}N+m-1\\ N\end{array})\propto \frac{{N}^{m-1}}{(m-1)!},$$

hence the method scales polynomially, with the order of the polynom depending on the number *m* of different numbers *n*_*kl*_. Please note that the number m, which is the number of involved spin matrices *σ*_*kl*_, does not have to be identical to (*d* + 1)^2^, the total number of individual spin matrices for a (*d* + 1)-level system. It can be lower if additional symmetries apply (see below).

The basis states defined in equation (11) are orthogonal with respect to the Hilbert-Schmidt inner product. Equation (14) allows to eliminate one of the *n*_*kl*_ coefficients. We usually eliminate *n*_00_, the number of multi-level systems sitting in the ground state.

As an illustration we consider *N* = 2 two-level systems: The permutation symmetric two-level system states are described by three numbers *n*_11_, *n*_10_, *n*_01_ (omitting *n*_00_), the basis elements are

16 $$\hat{{\mathscr{P}}}[{n}_{11},{n}_{10},{n}_{01}]={\mathscr{S}}\,{\sigma }_{11}^{\otimes {n}_{11}}{\sigma }_{10}^{\otimes {n}_{10}}{\sigma }_{01}^{\otimes {n}_{01}}{\sigma }_{00}^{\otimes {n}_{00}},$$

where ${n}_{00}=N-{n}_{11}-{n}_{10}-{n}_{01}$. According to equation (15) this results in $$(\begin{array}{c}2+3\\ 2\end{array})=10$$ possible basis states. These 10 states are given in Table 1.

**Table 1:** All permutation symmetric basis states for 2 two-level systems. Swapping the indices $1\leftrightarrow 2$ leaves these states invariant.

$\hat{{\mathscr{P}}}\mathrm{[0,}\,\mathrm{0,}\,\mathrm{0]}={\sigma }_{00}^{1}{\sigma }_{00}^{2}$	$\hat{{\mathscr{P}}}\mathrm{[1,}\,\mathrm{0,}\,\mathrm{0]}={\sigma }_{11}^{1}{\sigma }_{00}^{2}+{\sigma }_{00}^{1}{\sigma }_{11}^{2}$
$\hat{{\mathscr{P}}}\mathrm{[2,}\,\mathrm{0,}\,\mathrm{0]}={\sigma }_{11}^{1}{\sigma }_{11}^{2}$	$\hat{{\mathscr{P}}}\mathrm{[0,}\,\mathrm{1,}\,\mathrm{0]}={\sigma }_{10}^{1}{\sigma }_{00}^{2}+{\sigma }_{00}^{1}{\sigma }_{10}^{2}$
$\hat{{\mathscr{P}}}\mathrm{[1,}\,\mathrm{1,}\,\mathrm{0]}={\sigma }_{11}^{1}{\sigma }_{10}^{2}+{\sigma }_{10}^{1}{\sigma }_{11}^{2}$	$\hat{{\mathscr{P}}}\mathrm{[0,}\,\mathrm{2,}\,\mathrm{0]}={\sigma }_{10}^{1}{\sigma }_{10}^{2}$
$\hat{{\mathscr{P}}}\mathrm{[0,}\,\mathrm{0,}\,\mathrm{1]}={\sigma }_{01}^{1}{\sigma }_{00}^{2}+{\sigma }_{00}^{1}{\sigma }_{01}^{2}$	$\hat{{\mathscr{P}}}\mathrm{[1,}\,\mathrm{0,}\,\mathrm{1]}={\sigma }_{11}^{1}{\sigma }_{01}^{2}+{\sigma }_{01}^{1}{\sigma }_{11}^{2}$
$\hat{{\mathscr{P}}}\mathrm{[0,}\,\mathrm{1,}\,\mathrm{1]}={\sigma }_{10}^{1}{\sigma }_{01}^{2}+{\sigma }_{01}^{1}{\sigma }_{10}^{2}$	$\hat{{\mathscr{P}}}\mathrm{[0,}\,\mathrm{0,}\,\mathrm{2]}={\sigma }_{01}^{1}{\sigma }_{01}^{2}$

The actions of all terms in a permutation symmetric master equation only connects these 10 basis states. This is an instructive example for understanding the basis, the reduction in complexity however is minimal for this case (10 states compared to 2^2.2^ = 16 states for the full approach).

The formulation of the polynomial scaling method in ref.^1^ is given in terms of the density matrix elements ${\mathscr{P}}[\{{n}_{kl}\}]$, which are recovered from the symmetrized Liouville space states introduced in equation (11) by

17 $${\bf{t}}{\bf{r}}[\hat{{\mathscr{P}}}[\{{n}_{kl}\}]\rho ]={\mathscr{P}}[\{{n}_{kl}\mathrm{\}].}$$

The symmetrized states $\hat{{\mathscr{P}}}[\{{n}_{kl}\}]$ and the associated density matrix elements ${\mathscr{P}}[\{{n}_{kl}\}]$ are the formal foundation for PsiQuaSP. However this formulation is not very intuitive and not useful for setting up a simulation. Therefore in ref.^1^ we have developed a graphical sketch representation for these elements. The sketches are relatively simple and give an intuitive picture of the processes in the master equation, see Fig. 1. PsiQuaSP is designed in a way that allows the user to translate these sketches directly into code. The user does not need to derive any equations of motion, which facilitates the usage and greatly speeds up code development time. However a basic understanding of the sketches as well as the principles of the permutation symmetry is crucial for a successful usage of PsiQuaSP.

The sketches are intended to visualize the physical processes associated with the different contributions in the master equation: We can e.g. derive the two-level system spontaneous emission contribution equation (7)

18 $${\partial }_{t}{\mathscr{P}}[{n}_{11},{n}_{10},{n}_{01}{]|}_{{D}_{1}(\rho )}=\frac{\gamma }{2}\mathrm{[2(}{n}_{11}+\mathrm{1)}{\mathscr{P}}[{n}_{11}+\mathrm{1,}{n}_{10},{n}_{01}]-\mathrm{(2}{n}_{11}+{n}_{10}+{n}_{01}){\mathscr{P}}[{n}_{11},{n}_{10},{n}_{01}\mathrm{]].}$$

This equation describes density decay as well as decay induced dephasing of quantum coherences.

The density matrix elements ${\mathscr{P}}[{n}_{11},{n}_{10},{n}_{01}]$ correspond to a quantum coherence/correlation for ${n}_{10},{n}_{01}\ne 0$, for ${n}_{10},{n}_{01}=0$ the element corresponds to the population of the *N* two-level system setup in a state with *n*_11_ excitations, a density. Generally for elements ${\mathscr{P}}[\{{n}_{kl}\}]$, if the numbers *n*_*kl*_ corresponding to flip operators $(i\ne j)$ are zero, then the element is a density, otherwise the element corresponds to a quantum coherence.

We visualize the decay process as arrows by drawing the four degrees of freedom *n*_00_, *n*_01_, *n*_10_ and *n*_11_ as bubbles, see Fig. 1a. The full sketch for the master equation for the Dicke Hamiltonian equation (6) and the two-level system spontaneous emission is shown in Fig. 1b. Figure 1c shows the sketch for the Λ setup defined by equations (9) and (10). Figure 1d corresponds to a three-level laser setup, which has further symmetries that lead to an additional reduction in degrees of freedom and thus also dimensionality/numerical complexity. For the three-level laser setup 4 coherence degrees of freedom (*n*_20_, *n*_21_, *n*_02_ and *n*_12_) are decoupled from the densities. Since these decoupled coherences only dephase and are not driven we can set them to zero. More specific, for every basis element $\hat{{\mathscr{P}}}[\ldots ]$ with a nonzero index *n*_20_, *n*_21_, *n*_02_ or *n*_12_ the corresponding density matrix element is always zero. For more information on the rules for constructing the sketches please refer to ref.^1^ and Section 5.

PsiQuaSP uses PETSc or dependent packages to compute all relevant density matrix elements

19 $${\bf{t}}{\bf{r}}[\hat{{\mathscr{P}}}[\{{n}_{kl}\}]\rho ],$$

which are represented by a single column vector in memory. The Liouville operator ℒ is thus represented as a matrix. PsiQuaSP provides all functionality to setup arbitrary master equations and observables, etc. that are compatible with the permutation symmetric scheme. The translation into the internal representation used for calculation is completely handled by PsiQuaSP. As a free bonus also master equations without permutation symmetry can be set up, since any system described by a finite dimensional Hilbert space can be represented as a single multi-level system.

## Using PsiQuaSP–Basic structure of the library

PsiQuaSP is designed in a way that provides maximal flexibility for setting up simulations. Therefore PsiQuaSP only provides setup routines, i.e. for constructing the density matrix and the Liouvillian ${\mathcal L}$. Furthermore it allows to define observables, distributions, correlation functions etc. and encapsulates them in a user friendly way. The numerical solution solely relies on PETSc, derived packages such as SLEPc and/or external packages that can be used with PETSc such as MUMPS, SuperLU, Metis/ParMetis, PTScotch and others^49–59^, like in other PETSc based libraries^8^. Getting to know all these packages requires a lot of time and effort, but the average user can use PsiQuaSP without knowing details about these additional packages. However we wish to encourage the readers of this report to get to know these packages and related numerical literature and find out what they can do in order to boost the performance of the application code. The right choice of method can reduce computing time by orders of magnitude, see Section  *Performance*. Since PETSc and most related packages are written in C the choice of language for PsiQuaSP is the C family. This is in contrast to the widely used quantum optical numerics package based on Python^6,7^, following a different approach in their architecture.

**Figure 2:**
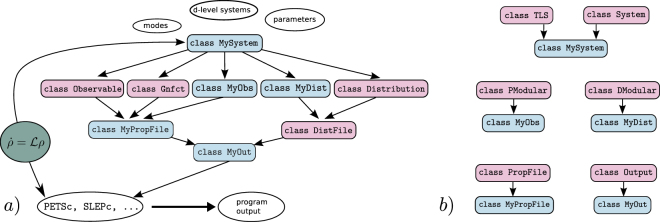
(**a**) Schematic representation of the general structure of a PsiQuaSP application code: contains all the relevant information about the system and is used to construct the master equation and the output. The master equation (green circle) is fed directly into the PETSc, SLEPc solvers, but also defines the overall system, Liouville space size, etc., which is represented by a double pointed arrow. The output is organized in three layers: The first layer consists of objects that can compute the desired properties of the system, like,  the correlation functions Gnfct and the custom types  and . The second layer groups these objects into output files, each managed by another object. The third layer consists of the  class, which groups all output files and provides a clean interface to PETSc. Classes that need to be derived from base classes have blue boxes, pink boxes indicate ready to use classes. (**b**) Base class diagram for the derived classes in (**a**). Only for  there are two possibilities:  for two-level system setups and  for all other purposes.

The heart of PsiQuaSP is the System class. The user first specifies whether two-, three- or (*d* + 1)-level systems are used and how many bosonic modes are required. For two-level systems the special TLS class provides further encapsulation and therefore simplification for standard two-level system Hamiltonians and dissipators. When using only the standard TLS class features there is actually no need for the sketch representation, one can directly translate a master equation of the form of equations (5), (8) and (7) into code. Only when considering multi-level systems and custom type Liouvillians the sketches are needed (explained in detail below). Based on the information on multi-level sytems and bosonic modes the System/TLS class provides initialization functions for the density matrix and Liouvillians–thus everything required for setting up the master equation. The Output class manages the output, which includes a set of user defined output files, containing observables, correlation functions, distributions, etc, see Fig. 2a. Please note that even though PsiQuaSP is intended and designed for solving permutationally symmetric master equations, the library is not limited to this application. It may also be used for efficient treatments of nonidentical multi-level systems as well as Hamiltonian diagonalizations.

Installation instructions for PsiQuaSP and PETSc are given in the README.md and INSTALL.md files in the PsiQuaSP folder. PsiQuaSP uses Doxygen commenting. Doxygen translates the comments in the source code into a structured website representation, which is extremely useful for getting to know the library. Read doc/README.md for further information.

In the following we will give a short introduction on how to set up a PsiQuaSP simulation. Many example source codes that explain a large portion of the PsiQuaSP functionality can be found in the example/directory in the PsiQuaSP directory. An overview over the available examples is given in Table 2.

**Table 2:** Overview over the example codes and the concepts explained/introduced in these examples.

	System, concepts, techniques
	Open Tavis-Cummings model, simple observables, distributions, time-integration
	ex1a with thermal bath, PETSc concepts, adaptive time integration, Dicke distribution
	Two-level laser, incoherent pump, custom observables
	Direct steady state/null space computation using SLEPc Krylov-Schur algorithm
	Two-level laser with Non-RWA terms
	Lambda system setup, multi-level system usage
	Three-level laser
	Phononlaser/Lasercooling setup, custom Liouvillians
	Same as ex3a, using ParMETIS graph partitioning to exploit *U*(1) symmetry, leading to a reduction from *N* ^8^ to ~*N* ^7^

requires an additional SLEPc installation and for ex5 it is necessary to build PETSc with the  flag.

### Example: Open Tavis-Cummings relaxation

**Figure 3:**
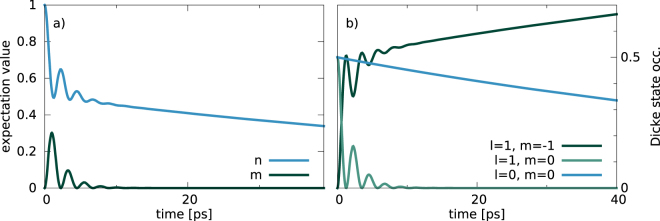
Using the code of example/ex1b: (**a**) mean excitation in the two-level systems $n=\langle {J}_{11}\rangle$ and mean photon number $m=\langle {b}^{\dagger }b\rangle$ for 2 two-level systems prepared in the state ${\mathscr{P}}\mathrm{[1,}\,\mathrm{0,}\,\mathrm{0;}\,\mathrm{0,}\,\mathrm{0]}$ − a single excitation in the two-level systems and zero photons. This corresponds to the entanglement distillation setup^76^. The bright superradiant states couple to the cavity mode and cause Rabi oscillations, while the dark subradiant state does not couple to the cavity and just decays via individual spontaneous emission^22,77^, c.f. equation (7). (**b**) Dicke state occupations $\langle |l,m\rangle \langle l,m|\rangle$: Temporal dynamics of the states of the superradiant subspace (green) vs. the single dark state in the subradiant subspace (blue). Parameters (as defined in Eq. (7) and Eq. (8) in a rotating frame): ${\omega }_{0}={\omega }_{11}$, $g=\mathrm{1.0\ }\,ps{}^{-1}$, $\gamma =\mathrm{0.01\ }\,ps{}^{-1}$, $\kappa =\mathrm{1.0\ }\,ps{}^{-1}$.

We consider the master equation defined by equations (5), (8) and (7) with graphical representation shown in Fig. 1a and b. This is a basic Tavis-Cummings model including individual spontaneous decay of the two-level systems and a cavity loss term. The example code computes the temporal dynamics of this master equation using direct Runge-Kutta time integration. The source code can be found in example/ex1a. example/ex1b solves the same equation with an adaptive step width Runge-Kutta and at the same time shows the application of more advanced PETSc routines. Since there is no pump term in this master equation the steady state is the ground state and we need to prepare the system initially in an excited state in order to observe nontrivial dynamics. The resulting temporal dynamics of this master equation are shown in Fig. 3.

*System/Master equation setup:* First we declare a derived class for the system under consideration:

class OTC: public TLS

{

public:

void Setup(Vec * dm, Mat * L);

};

This class just defines a setup function. This is the standard procedure in PsiQuaSP, for most cases user derived classes just define a setup function. We use the base class TLS, which provides enhanced tools for master equations only involving two-level systems. Here the setup function will create a vector Vec * dm and a matrix Mat * L, which are the density matrix and the Liouvillian of the system. PsiQuaSP uses a vectorized version of the master equation. The two types Vec and Mat are defined by PETSc. Both can be either serial or parallel, Mat is sparse by default, leading to efficient memory usage and reduction in computation time. If needed PETSc also provides dense matrix types, which can also be used with PsiQuaSP.

In the OTC::Setup(…) function we call the functions

TLSAdd(ntls,ntls,ntls,tlsenergy);

ModeAdd(m0 + 1, dm0, modeenergy);

PQSPSetup(dm,1,L);

to tell PsiQuaSP that we are considering nlts two-level systems and one bosonic mode with maximum Fock state m0. TLSAdd(…) adds the two-level system quantum numbers *n*_11_, *n*_10_ and *n*_01_, c.f. Fig. 1a and b. The three arguments ntls,ntls,ntls specify the maximum number for the three indices *n*_11_, *n*_10_, *n*_01_. This allows a truncation of the three individual quantum numbers. tlsenergy and modeenergy are the transition energies for exciting a two-level system and the photon energy. These energy parameters are usually written into the file headers and are needed for thermal state preparation and have no other purpose. They are independent of the parameters used for the equation of motion since a rotating frame representations might be used. After this the user needs to call PQSPSetup(), the setup function for all internal structures which creates the density matrix vector dm and the Liouvillian matrix L. Now the master equation needs to be specified. This is done by calling

AddTLSH0(*L, NULL, NULL, 1, domega_tls*PETSC_i);

AddTavisCummingsHamiltonianRWA(*L, NULL, NULL, 1, 0, gcouple*PETSC_i);

AddTLSSpontaneousEmission(*L, NULL, NULL, 1, gamma/2.0);

AddLindbladMode(*L, NULL, NULL, 1, 0, kappa/2.0);

Here each line adds the contributions of a different term of the master equation to the Liouvillian matrix L–the first two function calls add the von-Neumann part of the master equation given by equation (8) and the last two function calls add the two dissipator contributions equation (7). The sketch for AddTLSSpontaneousEmission(..) is shown in Fig. 1a and AddTavisCummingsHamiltonianRWA(..) is represented by the green arrows in Fig. 1b. Mode related Liouvillians like AddLindbladMode(…) are not represented with sketches. The sketch representing AddTLSH0(..) is given by the combination of the two sketches representing ${J}_{11}^{L}$, ${J}_{11}^{R}$ (see Section *Building arbitrary Liouvillians*). In this example we use a rotating frame representation and domega_tls is the detuning of the two-level systems from the cavity mode, on resonance domega_tls is equal to zero. As stated above, for this TLS class application there is no need to use sketches, one can directly implement the master equation–for this example using only four lines of code.

With this the setup of the master equation is complete. The preparation of the density matrix in an initial state as well as the setup of the observables and the whole program output are explained in detail in the Supplementary Information. There also the setup of three-level master equations is explained, which then can directly be generalized to arbitrary multi-level systems. However also the comments in the example source codes explain step by step the functionality of PsiQuaSP.

Examples using a variety of different master equations, custom observables, custom distributions, custom Hamiltonians and Liouvillians as well as other solution techniques and advanced, graph theory based reduction of degrees of freedom are provided in the example/ folder. Also in Section *Building arbitrary Liouvillians* as well as in the Supplementary Information there is further information on specific details on the setup of simulations. An overview of the current example codes and the explained concepts is given in Table 2.

## Template functions versus custom Liouvillians

PsiQuaSP has two types of possible usages. The first usage was presented in the previous section: Using ready-made functions for setting arrows of common Hamiltonians and Lindblad dissipators. Generally a single function call to one of these functions represents a single arrow in one of the sketches. First the user draws the sketch representation of the master equation and then directly translates the sketch into code. In the case of two-level systems a single function call is sufficient to set a Hamiltonian or dissipator contribution. The implemented contributions are shown in Table 3.

**Table 3:** Overview over the general ready-made Liouvillian setup functions of the System class.

Liouvillian	System function	Examples
$$H=\hslash {\omega }_{0}{b}^{\dagger }b$$		
$$H=\hslash {\omega }_{xx}{J}_{xx}$$		
$$H=\hslash g({J}_{xy}+{J}_{yx})({b}^{\dagger }+b)$$		
$$H=\hslash g({J}_{xy}{b}^{\dagger }+{J}_{yx}b)$$		
$$H=\hslash E({J}_{xy}{e}^{i\omega t}+{J}_{yx}{e}^{-i\omega t})$$		
$H=\hslash E(b{e}^{i\omega t}+{b}^{\dagger }{e}^{-i\omega t})$		**none**
$$D=\frac{\gamma }{2}{\sum }_{i}\mathrm{(2}{\sigma }_{xy}^{i}\rho {\sigma }_{yx}^{i}-{\sigma }_{yy}^{i}\rho -\rho {\sigma }_{yy}^{i})$$		
$$D=\delta {\sum }_{i}({\sigma }_{xy}^{z,i}\rho {\sigma }_{xy}^{z,i}-\rho )$$		
$$D=\frac{\kappa }{2}\mathrm{(2}b\rho {b}^{\dagger }-{b}^{\dagger }b\rho -\rho {b}^{\dagger }b)$$		
$$\begin{array}{c}D=\frac{\kappa }{2}((\bar{m}+\mathrm{1)(2}b\rho {b}^{\dagger }-{b}^{\dagger }b\rho -\rho {b}^{\dagger }b)+\bar{m}\mathrm{(2}{b}^{\dagger }\rho b-b{b}^{\dagger }\rho -\rho b{b}^{\dagger }))\end{array}$$		

Each arrow in the sketches of Fig. 1 can be set by a single function call to one of these functions. Hence a master equation represented by a sketch containing *n* arrows can be implemented by *n* function calls. Please look into the  class documentation to see the derived, specialized two-level system functions. The Hamiltonian contributions always refer to the $i/\hslash [\rho ,H]$ terms. Using ${\sigma }_{xy}^{z,i}=1/2({\sigma }_{xx}-{\sigma }_{yy})$.

In the second usage form the user defines elementary Liouville space operators and uses them to construct arbitrary master equations, observables, distributions, etc.: The permutation symmetric methodology is in principle applicable to *any* permutation symmetric quantum master equation and using the framework of PsiQuaSP in principle *any* quantum master equation in a number state representation can be solved (there is currently no support for coherent state basis etc.). Since we cannot provide template setup functions for every conceivable Liouvillian matrix another, more flexible approach is provided: In the second type of use case the user defines elementary Liouville operators, which act like

20 $${J}_{xy}\rho ={J}_{xy}^{L}\rho ,\quad \quad \rho {J}_{xy}={J}_{xy}^{R}\rho \mathrm{.}$$

Here we used the *L*, *R* algebra used in e.g. Liouville space calculations^72^: For any Hilbert space operator we define a Liouville space operator by distinguishing whether it acts on the left or right side of the density matrix, i.e. $A\rho ={A}^{L}\rho$ and $\rho A={A}^{R}\rho$. The operators ***A*** ^*L,R*^ are represented by matrices in PsiQuaSP, like every Liouville space operator. The setup of these elementary Liouville operators is done by first drawing a sketch for each needed operator and then adding all needed arrows by single function calls. Based on these elementary operators the user defines arbitrary interaction Hamiltonians and dissipators as well as custom observables, distributions, basis transformations etc. For instance using equation (20) the definition of a collective spontaneous emission Liouvillian from level *x* to level *y* is

21 $${\mathscr{D}}(\rho )=\frac{{\rm{\Gamma }}}{2}({J}_{yx}\rho {J}_{xy}-{J}_{xy}{J}_{yx}\rho -\rho {J}_{xy}{J}_{yx})\,=\,\frac{{\rm{\Gamma }}}{2}({J}_{yx}^{L}\cdot {J}_{xy}^{R}-{J}_{xy}^{L}\cdot {J}_{yx}^{L}-{J}_{yx}^{R}\cdot {J}_{xy}^{R})\rho ,$$

here the composite of the *R/L* operators, the operation ·, is performed by the standard matrix-matrix product and matrix addition, provided by the PETSc functions MatMatMult() and MatAXPY().

In summary the user first defines elementary matrices, e.g. for the ${J}_{xy}^{L,R}$ operators or any other operator, and then uses the PETSc matrix multiplication and addition functions to construct the desired Liouville operator. Additional details for this type of application case are explained in the next section.

## Building arbitrary Liouvillians

In this section we discuss a formalism for the setup of user defined Liouvillians which are consistent with the permutation symmetric method. We introduce separate setup functions for multi-level system and mode degrees of freedom e.g. for ${J}_{xy}^{L}$ or *b* ^*R*^. These elementary matrices can be used to construct more complicated operators such as ${J}_{xy}^{L}+{J}_{yx}^{R}$ and ${J}_{xy}^{L}{b}^{L}$ by using the PETSc matrix multiplication and addition. Defining setup functions for the mode degrees of freedom is straightforward and is based on textbook physics^73^. For the symmetric basis states of PsiQuaSP the treatment is a bit more difficult. The following discussion is technical since we want to explain the formal framework underlying PsiQuaSP in detail. The usage however is very simple, it results in drawing sketches and directly implementing each arrow by a single function call.

### Technical details

As defined in equation (19) PsiQuaSP uses an expansion of the density matrix in Liouville space. Expansion coefficients are calculated via the Hilbert-Schmidt inner product

22 $${\mathscr{P}}[\{{n}_{kl}\}]={\bf{t}}{\bf{r}}[\hat{{\mathscr{P}}}[\{{n}_{kl}\}]\rho ]$$

The actions of any operators *A*, *B* on the density matrix $A\rho B$ is handled by PsiQuaSP like applying these operators to $\hat{{\mathscr{P}}}[\{{n}_{kl}\}]$:

23 $${\bf{t}}{\bf{r}}[\hat{{\mathscr{P}}}[\{{n}_{kl}\}]A\rho B]={\bf{t}}{\bf{r}}[B\hat{{\mathscr{P}}}[\{{n}_{kl}\}]A\rho \mathrm{].}$$

Therefore we introduce a general recipe to construct arbitrary operators $B\hat{{\mathscr{P}}}[\{{n}_{kl}\}]A$ expressed in the permutation symmetric basis, for $A\rho B$ that live in the permutation symmetric subspace. Two steps are necessary: First we need to identify the elementary processes/Liouville operators and second we need to determine how to construct relevant operators, like e.g. a collective raising operator for a four-level system acting from the left. The permutation symmetry requires to include only processes acting *indistinguishably* on the left and/or right side of the density matrix. These elementary operators should be representable by arrows.

### Defining elementary processes/arrows

**Figure 4:**
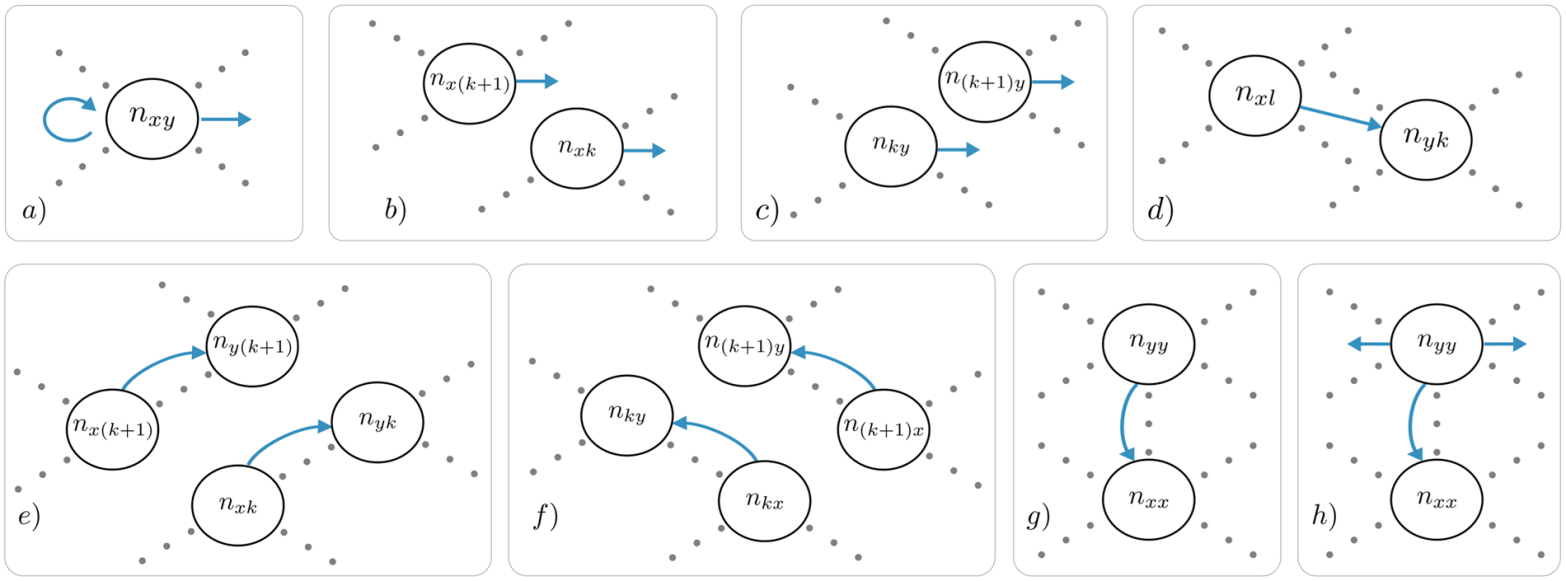
Modular sketches for multi-level systems: (**a**) The nonconnecting arrow can represent the phase oscillations arising from the self energy Hamiltonians (curved arrow) and it can describe dephasing (straight arrow). (**b** and **c**) the sketches corresponding to dephasing $\dot{\rho } \sim \rho {J}_{xx}$ and $\dot{\rho } \sim {J}_{yy}\rho$. One index each in the ${n}_{\ldots }$ numbers is fixed by the operators ${J}_{xx}^{R}$ and ${J}_{yy}^{L}$ and the whole operator is represented by the sum over the other, variable index *k*. (**d**) The connecting arrow can represent flip operators and density relaxation. (**e**) and (**f**) The arrows corresponding to the flip operators $\dot{\rho } \sim \rho {J}_{xy}$ and $\dot{\rho } \sim {J}_{xy}\rho$, c.f. equations (26) and (28). (**g**) The density relaxing arrow caused by an individual spontaneous emission like dissipator $\dot{\rho } \sim {\sum }_{i}{\sigma }_{xy}^{i}\rho {\sigma }_{yx}^{i}$. (**h**) The density relaxation arrow introduced in Fig. 1 (**a**) called by the function () consists of three arrows in the elementary picture, two nonconnecting and one connecting arrow.

Looking at the sketches in Fig. 1 we already see two general types of arrows: Connecting and nonconnecting arrows. A connecting arrow represents a coupling between two different symmetric basis states equation (11), corresponding to an in- or out-scattering process, and a nonconnecting arrow just acts on the state itself, leaving it unchanged. This is quite analogous to the actions of the interacting and non-interacting parts of a Hamiltonian acting on a Hilbert space state. In other words the symmetrized basis states equation (11) are eigenstates of the operators corresponding to the nonconnecting arrows. It turns out that these are the only possible two types. The general mathematical expressions are given by

24 $$\sum _{i}{\sigma }_{xx}^{i}\hat{{\mathscr{P}}}[\ldots ]{\sigma }_{yy}^{i}={n}_{xy}\hat{{\mathscr{P}}}[\ldots ]$$

for a single nonconnecting arrow and

25 $$\sum _{i}{\sigma }_{xy}^{i}\hat{{\mathscr{P}}}[\ldots ]{\sigma }_{kl}^{i}=({n}_{xl}+\mathrm{1)}\hat{{\mathscr{P}}}[\ldots {n}_{xl}+1\ldots {n}_{yk}-1\ldots ]{\rm{\Theta }}({n}_{yk}),$$

for a connecting arrow, where Θ(*n*) is equal to one for *n* > 0 and zero otherwise. Here we denote only the changed numbers *n*_*xl*_ and *n*_*yk*_, all other numbers remain unchanged. Applying the density matrix to these equations and taking the trace results again in the quantities for the equations of motion. Using the two types of arrows it is possible to construct every permutationally symmetric multi-level system Liouville space operator. The PsiQuaSP functions for adding one of the arrows to a given matrix are AddMLSSingleArrowNonconnecting(…) and AddMLSSingleArrowConnecting(…). The sketch representation for the two types is shown in Fig. 4a and d: Equation (24) for nonconnecting arrows can represent two different types of processes depending on the corresponding prefactor in the master equation. If the prefactor is imaginary the term corresponds to a Hamiltonian part *H*_0_, or if it is negative and real it corresponds to dephasing, caused e.g. by a dissipator (Fig. 4b and c). The two arrows are the looped and the outward pointing arrows in Fig. 4a). The connecting arrow equation (25) usually also represents two different processes: One sided flip operator actions arising from interaction Hamiltonians (Fig. 4e and f) and density relaxation caused by individual spontaneous emission, decay dissipators (Fig. 4g).

### Constructing physical operators

Looking at the collective flip operator acting from the right $\rho {J}_{xy}$

26 $$\begin{array}{rcl}{\bf{t}}{\bf{r}}[\hat{{\mathscr{P}}}[\ldots ]\rho {J}_{xy}]={\bf{t}}{\bf{r}}[{J}_{xy}\hat{{\mathscr{P}}}[\ldots ]\rho ] & = & {\bf{t}}{\bf{r}}[\sum _{i=1}^{N}{\sigma }_{xy}^{i}\hat{{\mathscr{P}}}[\ldots ]\sum _{k=0}^{d}{\sigma }_{kk}^{i}\rho ]\\  & = & \sum _{k}({n}_{xk}+\mathrm{1)}{\mathscr{P}}[\ldots {n}_{xk}+1\ldots {n}_{yk}-1\ldots ]{\rm{\Theta }}({n}_{yk}\mathrm{).}\end{array}$$

**Figure 5:**
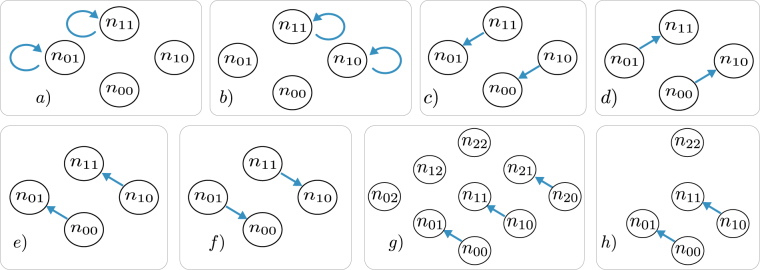
From (**a**) to (**f**): Sketches corresponding to ${J}_{11}^{L}$, ${J}_{11}^{R}$, ${J}_{10}^{R}$, ${J}_{01}^{R}$ and ${J}_{10}^{L}$, ${J}_{01}^{L}$ for two-level systems. When the operator acts on the left (right) side of the density matrix, it acts on the right (left) index of the *n*_*xy*_, c.f. equation (23). Two versions of the ${J}_{10}^{L}$ operators for the full and reduced three level system dynamics, c.f Fig. 1 (**c** and **d**).

Here *x* and *y* are set by the operator ***J***_*xy*_ and the whole operator is represented by the sum over all possible *k* values. Therefore a sum over all possible individual connecting arrows is required, see Figs 4e and 5c and d. Here in the second line we have inserted the Hilbert space identity for each individual (*d* + 1)-level system

27 $${I}^{i}=\sum _{k=0}^{d}{\sigma }_{kk}^{i}\mathrm{.}$$

The action of the ${\sigma }_{xy}^{i}$ matrices in equation (26) change each individual spin matrix ${\sigma }_{yk}^{i}$ into a spin matrix ${\sigma }_{xk}^{i}$. The $k$ sum of the ${\sigma }_{kk}^{i}$ matrices results in a sum over all possible $k$ indices in ${n}_{yk}$ and ${n}_{xk}$. In the last step we insert equation (25) and perform the trace operation. In this expression we see that the resulting matrix is sparse: The equation corresponds to the product of one row of the matrix with the column vector density matrix and thus there are at most $k$ nonzero entries in each row of this matrix.

The same operator acting from the left results in a sum over all possible left $k$ indices

28 $${\bf{t}}{\bf{r}}[\hat{{\mathscr{P}}}[\ldots ]{J}_{xy}\rho ]={\bf{t}}{\bf{r}}[\sum _{k}\sum _{i}{\sigma }_{kk}^{i}\hat{{\mathscr{P}}}[\ldots ]{\sigma }_{xy}^{i}\rho ]=\sum _{k}({n}_{ky}+\mathrm{1)}{\mathscr{P}}[\ldots {n}_{ky}+1\ldots {n}_{kx}-1\ldots ]{\rm{\Theta }}({n}_{yk}\mathrm{).}$$

These two operators can be implemented by repeatedly calling the AddMLSSingleArrowConnecting(…) function–once for every possible $k$ value, see Fig. 4e and f. The action of a collective projection or diagonal operator ${J}_{xx}$ is given by

29 $${\bf{t}}{\bf{r}}[\hat{{\mathscr{P}}}[\ldots ]\rho {J}_{xx}]={\bf{t}}{\bf{r}}[\sum _{k}\sum _{i}{\sigma }_{xx}^{i}\hat{{\mathscr{P}}}[\ldots ]{\sigma }_{kk}^{i}\rho ]=\sum _{k}{n}_{xk}{\mathscr{P}}[\ldots ]$$

and

30 $${\bf{t}}{\bf{r}}[\hat{{\mathscr{P}}}[\ldots ]{J}_{xx}\rho ]={\bf{t}}{\bf{r}}[\sum _{k}\sum _{i}{\sigma }_{kk}^{i}\hat{{\mathscr{P}}}[\ldots ]{\sigma }_{xx}^{i}\rho ]=\sum _{k}{n}_{kx}{\mathscr{P}}[\ldots ],$$

which can be implemented by repeatedly calling AddMLSSingleArrowNonconnecting(…) –again once for every possible $k$ value, see Fig. 4b and c. Having these steps in mind it is clear how to construct a general self energy Hamiltonian $\dot{\rho }\sim i/\hslash [\rho ,{H}_{0}]$ or a general individual dissipator:

31 $$D\rho =\frac{\gamma }{2}(\sum _{i}{\sigma }_{xy}^{i}\rho {\sigma }_{yx}^{i}-{J}_{yy}\rho -\rho {J}_{yy}\mathrm{).}$$

The first term is set by a single call to AddMLSSingleArrowConnecting(…), see equation. (25), and the second and third term are set as in equations 29 and 30. Please note that the possibility of a decoupling of some coherence degrees of freedom as in Fig. 1d is the main reason why PsiQuaSP does not provide generalized setup functions for operator actions of ${J}_{xy}$ and ${J}_{xx}$, since it would result in unnecessary numerical cost, if the decoupled basis elements were included. The other reason is that the elementary arrow representation also provides maximal freedom, whereas any encapsulation/facilitation would always be associated with a loss in generality.

The sketches for simple operators like ${J}_{xy}$ and ${J}_{xx}$ are easy to draw, see Fig. 5. Sketches corresponding to Liouville operators like ${J}_{xy}\rho {J}_{yx}$ or ${J}_{xy}^{n}\rho$ are more complicated and it is not recommended to implement them by hand as single operators. Rather we recommend to define the elementary operators like ${J}_{xy}$ and ${J}_{xx}$ and set the corresponding matrices. Then use the PETSc tools MatMatMult() and MatAXPY(…) to construct the combined operators. The following identities are useful for this case

32 $$A\rho B\,\stackrel{\wedge}{=}\,{A}^{L}\cdot {B}^{R}\rho ={B}^{R}\cdot {A}^{L}\rho ,\quad \quad AB\rho \,\stackrel{\wedge}{=}\,{A}^{L}\cdot {B}^{L}\rho ,\quad \quad \rho AB\,\stackrel{\wedge}{=}\,{B}^{R}\cdot {A}^{R}\rho ,$$

where the · operation is given be the MatMatMult() operation. The elementary Liouville space operators for the bosonic modes are set by calling the functions shown in Table 4.

**Table 4:** List off all available functions for setting elementary mode Liouvillians. The redundant functions allow faster and easier code development – actually all Liouvillians could be constructed from the first row.

*bρ*		*ρb*	
*b* ^†^ *ρ*		*ρb* ^†^	
*b* ^†^ *bρ*		*ρb* ^†^ *b*	
*bb* ^†^ *ρ*		*ρbb* ^†^	
*bρb* ^†^		*b* ^†^ *ρb*	

### Simple example

In example/ex4a we implement the phonon laser/laser cooling master equation from refs^74,75^, which represents a set of two-level systems coupled to a phonon mode and driven by an external laser, usually at the Stokes or anti-Stokes resonances

33 $$H=\hslash {\rm{\Delta }}{J}_{11}+\hslash {\omega }_{ph}{b}^{\dagger }b+\hslash g{J}_{11}(b+{b}^{\dagger })+\hslash E({J}_{10}+{J}_{01}\mathrm{).}$$

Here ${\rm{\Delta }}={\omega }_{11}-{\omega }_{L}$ is the detuning of the two-level systems from the driving laser. For positive detuning near the Stokes resonance this corresponds to laser cooling and for negative detuning at the anti-Stokes resonance this corresponds to phonon lasing. We include individual spontaneous emission and finite phonon lifetime

34 $${{\mathscr{D}}}_{1}(\rho )=\frac{\gamma }{2}\sum _{i}({\sigma }_{01}^{i}\rho {\sigma }_{10}^{i}-{\sigma }_{11}^{i}\rho -\rho {\sigma }_{11}^{i}),\quad \quad {{\mathscr{D}}}_{2}(\rho )=\frac{\kappa }{2}(b\rho {b}^{\dagger }-{b}^{\dagger }b\rho -\rho {b}^{\dagger }b\mathrm{).}$$

In this example six two-level system operators are required to construct the Hamiltonian or rather the von-Neumann part in the master equation: ${J}_{11}^{L,R}$, ${J}_{10}^{L,R}$ and ${J}_{01}^{L,R}$. Each of these matrices is defined by two calls to AddMLSSingleArrowNonconnecting(…) for ${J}_{11}^{L,R}$ and AddMLSSingleArrowConnecting(…) for ${J}_{10}^{L,R}$ and ${J}_{01}^{L,R}$. The sketches for these matrices are shown in Fig. 5. From these matrices and the respective phonon matrices all Hamiltonians are constructed.

## Performance

Two main advantages of PsiQuaSP are the reduction of complexity due to the symmetrized basis states and the manifold of solvers provided through PETSc and e.g. SLEPc.

### Overall complexity

**Figure 6:**
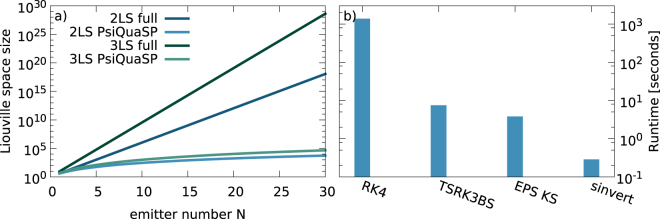
(**a**) The size of the Liouville space of the full exponential approach ($\sim {(d+\mathrm{1)}}^{N\cdot 2}$) for *N* (*d* + 1)-level systems vs. the permutation symmetric PsiQuaSP approach (equation (15)) for two-level systems and three-level systems. The required storage space and computation time scale at least linearly with this size. (**b**) Runtime comparison between different solution methods for steady state calculations for a two-level laser setup: Fixed time step fourth order Runge-Kutta (RK4), adaptive time step Runge-Kutta (TSRK3BS), SLEPc Krylov-Schur null space computation (EPS KS) and SLEPc Krylov-Schur null space computation with exact shift and invert spectral transformation (sinvert). Please refer to the PETSc and SLEPc documentation for details on these solvers.

In Fig. 6a the number of basis elements of the density matrix for the full exponential density matrix is compared to the polynomial, symmetrized PsiQuaSP density matrix for two- and three-level systems. This corresponds to the overall complexity since both the storage requirement and the number of coupled equations scale like the number of basis elements.

### Steady state computation

In Fig. 6b the convergence time for steady state calculations for a two-level laser as discussed in ref.^1^ and implemented in the examples example/ex2a and example/ex2b for different solvers is shown: The fixed time step fourth order Runge-Kutta is by far the slowest solver.

The adaptive time step and the direct null space computation using the SLEPc package outperform the fixed time step Runge-Kutta. The speedup of the shift and invert spectral transformation solver^50,51,53,54^ compared to the fourth order Runge-Kutta method is almost a factor of 5000. Please note that these numbers and the relative performance of the solvers are parameter and system size dependent, it is possible to find examples where the difference is even higher but it is also possible to find examples where the difference is less pronounced. Especially for iterative solvers like the SLEPc Krylov-Schur eigenvalue solver convergence time is highly dependent on the spectrum of the matrix and on chosen solver specific parameters. Please refer to the PETSc and SLEPc documentations for the specifics of these methods^49–54^.

## Summary

We have introduced a library that enables the setup of master equations for identical multi-level systems. The library provides ready-made setup functions for density matrices as well as Liouville operators. The design of these functions is centered around the sketch representation of the Liouville operators or master equation introduced in ref.^1^. This has the advantage that implementing an arbitrary master equation does not require calculating any equations of motion but can be done by directly implementing the sketches. There is a simplified usage for two-level systems and ready-made Liouvillian setup routines and an advanced usage where the user can construct arbitrary permutation symmetric Liouvillians from simple sketches.

### PsiQuaSP code

The code of the PsiQuaSP library can be found on GitHub: https://github.com/modmido/psiquasp.

## Electronic supplementary material

Supplementary Information
