# Supplementary material for: PsiQuaSP–A library for efficient computation of symmetric open quantum systems
Source: Sci Rep. 2017 Nov 24;7:16304. doi: 10.1038/s41598-017-16178-8 (PMC5701261; doi:10.1038/s41598-017-16178-8)
Supplement: Supplementary file 1 — Supplementary Information [file 41598_2017_16178_MOESM1_ESM.pdf]

PsiQuaSP – A library for efficient computation of symmetric open quantum systems

Michael Gegg[1,*] **and Marten Richter**[1]
Institut für Theoretische Physik, Nichtlineare Optik und Quantenelektronik, Technische Universität Berlin, Hardenbergstr. 36, EW 7-1, 10623 Berlin, Germany
michael.gegg@tu-berlin.de

S1 Supplementary Information

S1.1 Additional information for the Open Tavis-Cummings relaxation

In Section 3 in the main text we set the master equation for the open Tavis-Cummings model. To start a simulation initial conditions have to be specified. Here we prepare the system in an excited state:

```
PetscInt qnumbers [5] = {n11,n10,n01,mket,mbra};
DMWritePureState(*dm,qnumbers);
```

The `qnumbers` array contains the quantum numbers of the desired state. This setup function can prepare the density matrix in any of the permutation symmetric basis states (equation (11)). However only for `n10`, `n01` equal to zero and `mket` = `mbra` the state corresponds to a physically meaningful population. This function addresses the different quantum numbers in the order they have been set: The `TLSAdd(...)` function call adds the two-level system quantum numbers in the order n_{11}, n_{10}, n_{01} and the function `ModeAdd(...)` always adds first the ket and then the bra quantum number of |`mket`⟩⟨`mbra`|. If we had added another mode via two successive calls to `ModeAdd(...)` we would address an individual state with an array like

```
PetscInt qnumbers [7] = {n11,n10,n01,m0ket,m0bra,m1ket,m1bra};
```

PsiQuaSP internally labels the modes with numbers starting from 0 in the order of creation.
To create an object of the system specification class `OTC` that we just defined, we call e.g. in the main routine

```
OTC otc;
otc.Setup(&dm,&L);
```

The `otc` object has two purposes: It creates all ingredients to the master equation and after successful setup it contains all necessary information about the system. Afterwards the object is used to build observables and to specify the output data.

S1.1.1 Defining the program output

The expectation value of a collective operator like J_{11} (equation (3)), which represents the mean occupation of the excited states of all two-level systems

$$\langle J_{11}\rangle = \mathbf{tr}[J_{11}\rho] = \sum_{n=0}^{N}\sum_{m} n\mathcal{P}[n,0,0;m,m] \tag{S1}$$

can be defined with the command

```
Observable *pdens11 = new Observable();
MLSDim n11 (1,1);
pdens11->SetupMlsOccupation(otc,n11);
```

Here `n11` is an identifier referring to the n_{11} degree of freedom and the function `SetupMlsOccupation()` can define all $\langle J_{kk}\rangle$ observables for arbitrary multi-level systems

$$\langle J_{kk}\rangle = \sum_{n_{kk}=0}^{N}\sum_{\dots} n_{kk}\mathcal{P}[\dots n_{kk}\dots], \tag{S2}$$

where the second sum runs over all indices describing a density i.e. a partial trace. The `MLSDim` and `ModeDim` classes provide a way to access different degrees of freedom within the application code. Output files that print observables,

distributions etc. at every nth time step are also managed by classes. The printout interval n is equal to 30 by default and can be changed with the `-tev_steps_monitor newvalue` command line option. For printing observables like the expectation value of J_{kk} into files the user creates a derived class like

```
class ObservablesFile:  public PropFile
{
 public:
 void SetupMyObsFile(OTC * otc, std::string name);
};
```

As in the `OTC` class only the definition of a setup function is required. `name` is the name for the output file. This class is derived from the `PropFile` class. Classes derived from this class allow the user to print an arbitrary number of user specified properties that are related to operator expectation values. This includes standard (already implemented) observables of the `Observable` class, correlation functions $g^{(n)}(\tau)$ (`Gnfct` class) and user defined custom observables (`PModular` class). Within this setup function we create the `Observable` object as above and add it to the output file with the command

```
AddElem(pdens11,"<J_11>");
```

The second argument is the name of this quantity in the header of the file. This observables file including an arbitrary number of other user specified output files is bundled into the `MyOut` class

```
class MyOut:  public Output
{
 public:
 void SetupMyOut(OTC * system);
};
```

The setup function includes the following function calls

```
ObservablesFile *obsfile = new ObservablesFile;
obsfile->SetupMyObsFile(system,"observables.dat");
AddOFile(obsfile);
```

We can specify an arbitrary number of different output files for customized purposes by either providing multiple setup functions in the `ObservablesFile` class or deriving a new class with a single setup function for each file e.g. `ObservableFile1`, `ObservableFile2` .... The `DistFile` class is used for number state distributions of the modes and the multi-level systems, as well as more complicated (also custom made) distributions like the `DickeDistribution`.
As for the `OTC` class e.g. in the main file we need to call

```
MyOut *out = new MyOut;
out->SetupMyOut(&otc);
```

These function calls create the whole output structure of the program bundled into one object. Generally PsiQuaSP provides functionality for setting up vectors and matrices and to create the output object (`out`). These three objects (`Vec`, `Mat` and `MyOut`) then provide the input fed into the PETSc (SLEPc, ...) solution routines.

S1.1.2 Observables as linear functionals

Please note that PETSc vectors can also represent observables, since a vector defines a functional of the vector space through the inner product. For example the trace operation is just the vector where every density degree of freedom is set to one and all other entries are set to zero. Therefore any computation of an observable can be defined as a PETSc vector (vector of dual space). This linear functional is subsequently applied to the density matrix via a scalar product using the PETSc routine `VecDot(...)`: Defining a custom observable $\langle \hat{O} \rangle$ usually is done by setting the matrix for the Liouvillian corresponding to the action of $\hat{O}\rho$, multiplying it with the trace vector and storing the resulting vector/linear functional. The computation of the observable is then calculated

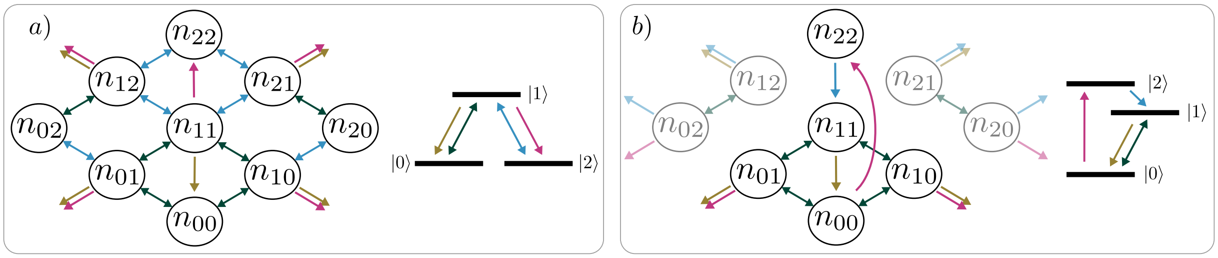

Figure S1. Same as Fig. 1 c) and d): a) Λ-system setup of equations (9), (10): Two different interactions from equation (9) (green,blue) and two different spontaneous emission processes from equation (10) (yellow,purple). b) Three-level laser setup (Ref. 1): Population mechanism through incoherent driving (pink,blue), coupling to the lasing mode (green) and spontaneous emission into nonlasing modes (yellow). Four coherence degrees of freedom (n_{20}, n_{21}, n_{02} and n_{12}) are decoupled from the densities.

using the scalar product of this vector with the density matrix vector (`VecDot(...)`). This is shown in `example/ex2a`.

S1.1.3 Solution with PETSc

The numerical solution is handled by PETSc, in this example we use simple time integration using a normal fourth order Runge-Kutta in `example/ex1a` and an adaptive time step Runge-Kutta in `example/ex1b`. The basic setup of a time integration using PETSc is as follows:

```
TS ts;
TSCreate(PETSC_COMM_WORLD,&ts);
TSSetType(ts,TSRK);
```

This creates the PETSc time stepper context `TS` and sets it to Runge-Kutta. `PETSC_COMM_WORLD` is the PETSc MPI communicator. PsiQuaSP is fully parallelized by default by using the PETSc routines, but it can of course always be run on a single processor. With the commands

```
TSSetRHSFunction(ts,NULL,TSComputeRHSFunctionLinear,NULL);
TSSetRHSJacobian(ts,L,L,TSComputeRHSJacobianConstant,NULL);
```

we tell PETSc that the right hand side of the differential equation ($\dot{\rho} = \mathcal{L}\rho$) is given by a constant matrix and that this matrix is the `L` matrix. The output of the PETSc time steppers is handled by a monitor function, which is a function with a defined interface that PETSc calls at every integration step:

```
TSMonitorSet(ts,MyOut::TEVMonitor,out,NULL);
```

The function `MyOut::TEVMonitor` is the monitor function for time integration in PsiQuaSP. It prints a single line at every nth time step into each specified output file by computing all user specified observables and distributions in each individual file (`-tev_steps_monitor newvalue` to change n). The command

```
TSSolve(ts,dm);
```

solves the time dynamics, `dm` contains always the current time step density matrix. In Fig. 3 a) in the main text the mean excitation in the two-level systems and the mode during this relaxation is shown: Initially the dynamics are fast due to Rabi oscillations between bright Dicke states and the mode. Afterwards the dynamics is governed by the slow, monotonous spontaneous emission, since only the dark Dicke states remain excited. In Fig. 3 b) the population in these Dicke states is shown.

S1.2 Example 2: Three-level systems using `System` class

In the two-level system example we used the base class `TLS`. For three- and general multi-level systems specialized classes are not provided, instead there is the multi purpose class `System` (the base class of `TLS`). In Figs. S1 a) and b) two different three-level system sketches are shown: Fig. S1 a) connects all degrees of freedom while in Fig. S1

b) four degrees of freedom can be eliminated, resulting in a $\sim N^4$ scaling instead of an $\sim N^8$ scaling for Fig. S1 a). The decoupling of some basis states and the resulting reduction in degrees of freedom is the main reason why PsiQuaSP does not provide specialized classes for multi-level systems. In the following we will discuss both examples simultaneously to illustrate the differences, simplifications arising with the decoupling in the sketches.
For the two-level system example we called `TLSAdd(a,b,c,energy)` which uses internally

```
MLSAddDens(n11,a+1,energy);
MLSAddPol(n10,b+1);
MLSAddPol(n01,c+1);
```

where `MLSAddDens(...)` adds a density degree of freedom, corresponding to a quantum number n_{xx}, and `MLSAddPol(...)` adds a polarization degree of freedom, corresponding to a quantum number n_{xy}, $x \neq y$. Thus the degrees of freedom for three level systems (Fig. S1 a)) are set with the function calls (without truncating basis states)

```
MLSAddDens(n22,n+1,energy2);
MLSAddPol(n21,n+1);
MLSAddPol(n20,n+1);
MLSAddPol(n12,n+1);
MLSAddDens(n11,n+1,energy1);
MLSAddPol(n10,n+1);
MLSAddPol(n02,n+1);
MLSAddPol(n01,n+1);
```

For the reduced dynamics of the three-level laser of Fig. 1 d) all necessary degrees of freedom can be set with just the function calls

```
MLSAddDens(n22,n+1,energy2);
MLSAddDens(n11,n+1,energy1);
MLSAddPol(n10,n+1);
MLSAddPol(n01,n+1);
```

Here `n` represents the number of three-level systems in the particular setup. This number can also be lower than the number of treated three-level systems, which corresponds to a truncation of the number of three-level system basis states. A truncation should always be tested if it is applicable in the given situation (parameter dependent), but it can reduce the numerical cost considerably (Example: strong dephasing in driven systems can reduce the number of needed offdiagonals (`nxy`) considerably). The `nxy` objects are again the `MLSDim` identifiers and are created with e.g.

```
MLSDim n21 (2,1);
```

As in the two-level system example, after setting all multi-level system degrees of freedom the user adds bosonic modes with the command

```
ModeAdd(m0+1,dm0,modeenergy);
```

`ModeAdd(...)` calls have to be after the `MLSAdd(...)` calls, PsiQuaSP returns an error message if these routines are not called in the right order. Setting e.g. the spontaneous emission dissipator between levels $1-0$ for Fig. S1 a) is done with

```
AddLindbladRelaxMLS(L,NULL,NULL,1,n11,n00,gamma/2.0);
AddLindbladDephMLS(L,NULL,NULL,1,n10,gamma/2.0);
AddLindbladDephMLS(L,NULL,NULL,1,n01,gamma/2.0);
AddLindbladDephMLS(L,NULL,NULL,1,n21,gamma/2.0);
AddLindbladDephMLS(L,NULL,NULL,1,n12,gamma/2.0);
```

For the three-level laser of Fig. S1 b) the spontaneous emission contribution simplifies to

```
AddLindbladRelaxMLS(L,NULL,NULL,1,n11,n00,gamma/2.0);
AddLindbladDephMLS(L,NULL,NULL,1,n10,gamma/2.0);
AddLindbladDephMLS(L,NULL,NULL,1,n01,gamma/2.0);
```

The parameter `gamma/2.0` is the same parameter as in equation (7) and each function call corresponds to exactly one arrow in the sketches. Incoherent pumping is added by calling

```
AddLindbladRelaxMLS(L,NULL,NULL,1,n00,n22,pump/2.0);
```

and the respective calls to `AddLindbladDephMLS()`. The interaction of the three-level systems with the mode for Fig. S1 a) is included by calling

```
AddMLSModeInt(AA,NULL,NULL,1,n20,n21,mbra,-gcouple*PETSC_i);
AddMLSModeInt(AA,NULL,NULL,1,n10,n11,mbra,-gcouple*PETSC_i);
AddMLSModeInt(AA,NULL,NULL,1,n00,n01,mbra,-gcouple*PETSC_i);
AddMLSModeInt(AA,NULL,NULL,1,n02,n12,mket,gcouple*PETSC_i);
AddMLSModeInt(AA,NULL,NULL,1,n01,n11,mket,gcouple*PETSC_i);
AddMLSModeInt(AA,NULL,NULL,1,n00,n10,mket,gcouple*PETSC_i);
```

and for the three-level laser of Fig. S1 b) this is done by omitting the arrows of the disconnected part of the sketch:

```
AddMLSModeInt(AA,NULL,NULL,1,n10,n11,mbra,-gcouple*PETSC_i);
AddMLSModeInt(AA,NULL,NULL,1,n00,n01,mbra,-gcouple*PETSC_i);
AddMLSModeInt(AA,NULL,NULL,1,n01,n11,mket,gcouple*PETSC_i);
AddMLSModeInt(AA,NULL,NULL,1,n00,n10,mket,gcouple*PETSC_i);
```

`mket` and `mbra` are the identifiers for the mode degrees of freedom and are created by calling

```
ModeDim mket (0,photonnumber);
ModeDim mbra (1,photonnumber);
```

`photonnumber` is the index of the mode. Modes are numbered internally starting from zero in the order they are created with an `AddMode()` call. Hamiltonian contributions that change the right index of the `MLSDim` and/or act on the bra side of the mode expansion come with a minus sign. This stems from the commutator in the von-Neumann part of the quantum master equation, see section 5 for more details. The generation of the output as well as the solution stage is completely analogous to the two-level system example, see Section S1.1.

References

1. Gegg, M. & Richter, M. Efficient and exact numerical approach for many multi-level systems in open system CQED. *New J. Phys.* **18**, 043037 (2016).
